# Fluorescence via Reverse Intersystem Crossing from Higher Triplet States in a Bisanthracene Derivative

**DOI:** 10.1038/s41598-017-05007-7

**Published:** 2017-07-06

**Authors:** Tohru Sato, Rika Hayashi, Naoki Haruta, Yong-Jin Pu

**Affiliations:** 1 Department of Molecular Engineering, Graduate School of Engineering, Kyoto University, Nishikyo-ku, Kyoto 615-8510 Japan; 2 Unit of Elements Strategy Initiative for Catalysts & Batteries, Kyoto University, Nishikyo-ku, Kyoto 615-8510 Japan; 3 Undergraduate School of Industrial Chemistry, Faculty of Engineering, Kyoto University, Sakyo-ku, Kyoto 606-8501 Japan; 4 Graduate School of Organic Materials Science, Yamagata University, Yonezawa, 992-8510 Yamagata Japan; 5 PRESTO (Sakigake), JST, Kawaguchi, Saitama 332-0012 Japan

## Abstract

To elucidate the high external quantum efficiency observed for organic light-emitting diodes using a bisanthracene derivative (BD1), non-radiative transition processes as well as radiative ones are discussed employing time-dependent density functional theory. It has been previously reported that the observed high external quantum efficiency of BD1 cannot be explained by the conventional thermally activated delayed fluorescence involving T_1_ exciton nor triplet-triplet annihilation. The calculated off-diagonal vibronic coupling constants of BD1, which govern the non-radiative transition rates, suggest *a fluorescence via higher triplets* (*FvHT*) *mechanism*, which entails the conversion of a high triplet exciton generated during electrical excitation into a fluorescent singlet exciton. This mechanism is valid as long as the relaxation of high triplet states to lower states is suppressed. In the case of BD1, its pseudo-degenerate electronic structure helps the suppression. A general condition is also discussed for the suppression of transitions in molecules with pseudo-degenerate electronic structures.

## Introduction

Thermally activated delayed fluorescence (TADF) has attracted significant attention as the emission mechanism in molecules used in organic light-emitting diodes (OLEDs)^1^. Although the phenomenon of TADF has been known for a long time, its application in OLEDs was first reported by Endo *et al*. in 2009^2^. TADF OLEDs utilize fluorescence via reverse intersystem crossing (RISC) from the triplet state, T_1_, generated during electrical excitation, as well as fluorescence from the singlet excited state, S_1_, generated during electrical excitation.

In order to make RISC possible in a molecule, the energy difference between S_1_ and T_1_, Δ*E*_ST_, must be small enough that the RISC energy barrier can be overcome through thermal excitation. Δ*E*_ST_ can be written as

1 $${\rm{\Delta }}{E}_{{\rm{ST}}}=2J=2\iint {{\psi }}_{{\rm{HO}}}^{\ast }({{r}}_{1}){\psi }_{{\rm{LU}}}({{r}}_{1})\frac{1}{{r}_{12}}{\psi }_{{\rm{HO}}}({{r}}_{2}){\psi }_{{\rm{LU}}}^{\ast }({{r}}_{2}){d}^{3}{{r}}_{1}{d}^{3}{{r}}_{2},$$

where *J* is an exchange integral, and *ψ*_HO_(*r*) and *ψ*_LU_(*r*) denote the HOMO and LUMO, respectively. Based on this equation, Endo *et al*. proposed a design principle to reduce Δ*E*_ST_^2^, i.e. candidates for TADF molecules must be donor-acceptor systems with small overlap between the HOMO and LUMO.

Based on this design principle for emitting molecules, a number of TADF OLEDs have exhibited very high external quantum efficiencies (EQEs). For example, a phenoxazine derivative, PXZ-TRZ, exhibits an EQE of 12.5% (photoluminescence quantum efficiency (PLQE): 65.7%)^3^, a carbazolyl dicyanobenzene derivative, 4CzIPN, exhibits an EQE of 19.3% (PLQE: 94%)^4^, a triazine derivative, CC2TA, exhibits an EQE of 11% (PLQE: 62%)^5^, a spiro bifluorene derivative, Spiro-CN, exhibits an EQE of 4.4% (PLQE: 27%)^6^, and an acridine derivative, ACRFLCN, exhibits an EQE of 10.1% (PLQE: 67%)^7^. Recently, Kaji *et al*. reported a triazine derivative, DACT-II, exhibiting an extremely high EQE of 41.5% (PLQE:100%)^8^. These results demonstrate the success of this design principle.

However, this design principle also has certain drawbacks: (1) the small overlap between the HOMO and LUMO leads to suppression of the oscillator strength^9, 10^, and (2) TADF OLEDs exhibit broad emission wavelengths because of charge-transfer (CT) excitation. In order to overcome these drawbacks, Sato *et al*. proposed other concepts for emitting molecules in OLEDs, viz. symmetry-controlled TADF (SC-TADF) and inverted singlet and triplet (iST) structure, wherein fluorescence via RISC from triplet states higher than T_1_ is utilized based on the selection rules of transition dipole moment (TDM) and spin-orbit coupling (SOC)^11^. The order of the preferable point groups for realizing SC-TADF and iST is as follows:

2 $$\begin{array}{c}{D}_{6h} > {O}_{h} > {I}_{h}={D}_{4h} > {D}_{2h} > {D}_{3h} > {T}_{d} > {C}_{i}={C}_{2h} > {D}_{2d}={C}_{4v} > {D}_{2}\\ \quad \quad \quad \quad \quad \,\,\,\,\,=\,{C}_{2v} > {C}_{3v} > {C}_{s}={C}_{1}.\end{array}$$

These mechanisms are unlike TADF because they enable us to (1) use candidates not belonging to the donor-acceptor type and (2) induce RISC without thermal excitation. Uejima *et al*. and Sato *et al*. have already designed and proposed iST molecules based on anthracene^9, 10^ and perylene derivatives^11^, respectively.

The concepts of SC-TADF and iST require a high molecular symmetry group. However, even for asymmetric molecules, RISC via higher T_*n*_ is possible as long as undesirable interactions are suppressed. Recently, a phenothiazin-benzothiadiazole derivative, PTZ-BZP, used as a fluorescent OLED exhibited a high EQE of 1.54% (PLQE:16%)^12^, which was attributed to fluorescence via RISC from T_3_ based on the energy gap law. Sato also proposed that the high EQE in PTZ-BZP, which is an asymmetric molecule, can be attributed to suppressed radiative and non-radiative transitions from triplet states higher than T_1_ to lower triplet states because of small overlap densities in the pseudo-degenerate electronic structure as well as the small energy gap between the relevant triplet and singlet states^13^.

The overlap density *ρ* ^*mn*^ between the *N*-electron wave functions of electronic states Ψ_*m*_ and Ψ_*n*_ is defined by

3 $${\rho }^{mn}({{r}}_{i})\,:=N\int \cdots \int {{\rm{\Psi }}}_{m}^{\ast }({{R}}_{0},{r}){{\rm{\Psi }}}_{n}({{R}}_{0},{r}){d}^{4}{{x}}_{1}\cdots {d}^{4}{{x}}_{i-1}d{s}_{i}{d}^{4}{{x}}_{i+1}\cdots {d}^{4}{{x}}_{N},$$

where *R*_0_ denotes a reference nuclear configuration, and *x*_*i*_ = (*r*_*i*_, *s*_*i*_) with spatial coordinate *r*_*i*_ and spin coordinate *s*_*i*_ for electron *i* ^9, 10^. Hereafter *r*_*i*_ is simply denoted as *r*. *ρ* ^*mn*^(*r*) is sometimes called a transition density^14^, especially within the orbital approximation. For example, in the case of the HOMO-LUMO transition, it is equal to HOMO-LUMO overlap density. The overlap density is related to the rate constants of radiative and non-radiative transitions as follows.

The rate constant of the radiative transition between electronic states *m* and *n* depends on the square of TDM, *μ*_*mn*_, defined as

4 $${\mu }_{mn}\,:=\int \cdots \int {{\rm{\Psi }}}_{m}^{\ast }({{R}}_{0},{r})(\sum _{i}-e{{r}}_{i}){{\rm{\Psi }}}_{n}({{R}}_{0},{r}){d}^{4}{{x}}_{1}\cdots {d}^{4}{{x}}_{N},$$

while that of the non-radiative transition via vibrational mode *α* depends on the square of off-diagonal vibronic coupling constant (VCC) ${V}_{\alpha }^{mn}$, defined as

5 $${V}_{\alpha }^{mn}\,:=\int \cdots \int {{\rm{\Psi }}}_{m}^{\ast }({{R}}_{0},{r}){(\frac{\partial \hat{H}}{\partial {Q}_{\alpha }})}_{{{R}}_{0}}{{\rm{\Psi }}}_{n}({{R}}_{0},{r}){d}^{4}{{x}}_{1}\cdots {d}^{4}{{x}}_{N},$$

where *e* denotes the elementary charge, $\hat{H}$ is a molecular Hamiltonian, and *Q*_*α*_ stands for a mass-weighted normal coordinate of mode *α* ^9, 10^. TDM *μ*_*mn*_ and off-diagonal VCC ${V}_{\alpha }^{mn}$ represent the strengths of radiative and non-radiative transitions, respectively. The relations of the transition rate constants to *μ*_mn_ and ${V}_{\alpha }^{mn}$ are described in more detail in Section S1 of the Supplemental Material. These rate constants were also derived on the basis of the Born–Oppenheimer approximation in past literatures^15, 16^. It should be noted that the present expression of the non-radiative rate constant is different from the other authors’^15, 16^. Eq. (5) is based on the crude adiabatic approximation^17^.

TDM *μ*_*mn*_ can be rewritten using the transition dipole moment density (TDMD) *τ*_*mn*_(*r*):

6 $${\mu }_{mn}=\int {\tau }_{mn}({r}){d}^{3}{r},$$

where the TDMD is defined by

7 $${\tau }_{mn}({r})\,:=-e{r}{\rho }^{mn}({r}\mathrm{).}$$

TDMD *τ*_*mn*_(*r*) illustrates the origin of TDM which causes radiative transition as a local picture. The detailed derivation of TDMD is shown in Section S2 of the Supplemental Material. The off-diagonal VCC ${V}_{\alpha }^{mn}$ can be exactly expressed using the off-diagonal vibronic coupling density (VCD) ${\eta }_{\alpha }^{mn}({r})$ ^9, 10^:

8 $${V}_{\alpha }^{mn}=\int {\eta }_{\alpha }^{mn}({r}){d}^{3}{r},$$

where the off-diagonal VCD is defined by

9 $${\eta }_{\alpha }^{mn}({r})\,:={\rho }^{mn}({r})\times {v}_{\alpha }({r}),$$

and the potential derivative *v*_*α*_(*r*) is defined by

10 $${v}_{\alpha }({r})\,:={(\frac{\partial u({r})}{\partial {Q}_{\alpha }})}_{{{R}}_{0}},\quad u({r})\,:=\sum _{A=1}^{M}-\frac{{Z}_{A}{e}^{2}}{4\pi {\varepsilon }_{0}|{r}-{{R}}_{A}|},$$

where *u*(*r*) is the attractive potential of a single electron due to all nuclei, and *R*_*A*_ and *Z*_*A*_ are the position and charge of nucleus *A*. VCD ${\eta }_{\alpha }^{mn}({r})$ illustrates the origin of VCC which gives rise to non-radiative transition as a local picture. The detailed derivation of VCD can be found in Section S2 of the Supplemental Material. Based on Eqs (6), (7), (8) and (9), both radiative and non-radiative transitions are suppressed when the overlap density *ρ* ^mn^(*r*) is small. As mentioned earlier, the unusual high EQE in PTZ-BZP^12^ has been successfully explained using the concept of the overlap density^13^.

**Figure 1:**
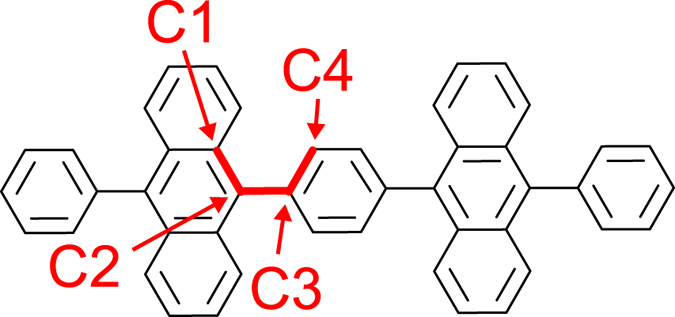
Bisanthracene derivative, BD1.

Recently, Hu *et al*. have observed blue-light emission in OLEDs using bisanthracene derivatives including 1,4-bis(10-phenylanthracene-9-yl)benzene (BD1) (see Fig. 1)^18^. Since the observed PLQE of BD1 is 14% in the neat film while the maximum EQE is 8.9% in the doped film, the emission is not conventional fluorescence using only singlet excitons. In other words, triplet excitons must contribute to the observed emission. However, the detailed mechanism of the observed high EQE is still unclear. Although one possible mechanism is the TADF, Δ*E*_ST_ between S_1_ and T_1_ was calculated to be too large to be overcome thermally^18^. Therefore, the conventional TADF involving T_1_ is invalid. Another possible mechanism is the triplet-triplet annihilation (TTA). In the TTA mechanism, 62.5% of generated excitons can be used as fluorescent singlet excitons at best. Hu *et al*. have reported that the observed PLQE of the neat film is 14%^18^. Using these values, the estimated upper-limit of the EQE is 1.8–3.5% even if the outcoupling efficiency is assumed to be 20–40%. However, the highest EQE value was reported to be 5.6% in the device employing a neat film of BD1 as an emitting layer. Therefore, we cannot explain the observed EQE on the basis of the TTA mechanism. The other mechanism is required to explain the observed EQE of BD1.

In this study, we theoretically investigate the mechanism of light emission from an OLED using a bisanthracene derivative, BD1, on the basis of the understanding of radiative and non-radiative transition processes. We propose *a fluorescence via higher triplets* (*FvHT*) mechanism to explain the high EQE in OLEDs using BD1. FvHT is valid as long as undesirable radiative and non-radiative transitions are suppressed. In this mechanism, the concept of overlap density plays a crucial role. In addition, we perform the analyses for electronic wave functions of BD1 in detail to obtain general design principles for the realization of the present mechanism.

## Results

The symmetry of the optimized structure for the ground state is not *D*_2*h*_ but *D*_2_ because the dihedral angle (C1–C2–C3–C4) between the anthracene and benzene moieties (see Fig. 1) is not the right angle, as shown in Figs. S1 and S2 in the Supplemental Material. Therefore, it is not suitable as an iST nor SC-TADF molecule, as expressed in Eq. (2) (see also Section S4 of the Supplemental Material).

**Figure 2:**
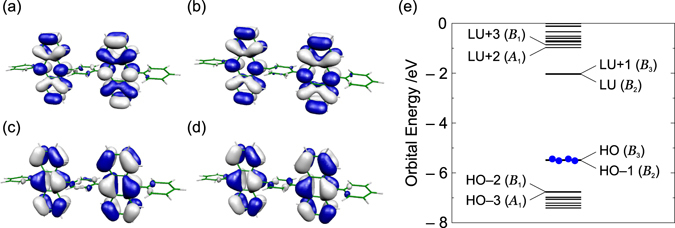
Frontier orbitals of BD1: (**a**) *B*_2_ LUMO, (**b**) *B*_3_ NLUMO, (**c**) *B*_3_ HOMO, and (**d**) *B*_2_ NHOMO. The isosurface value is 2.0 × 10^−2^ a.u. (**e**) Frontier orbital levels of BD1. It should be noted that the HOMO and next HOMO as well as the LUMO and next LUMO are pseudo-degenerate, respectively.

Frontier orbitals and their energy levels at the optimized structure of the ground state are shown in Fig. 2. The NHOMO *ψ*_NHO_ and HOMO *ψ*_HO_ as well as the NLUMO *ψ*_NLU_ and LUMO *ψ*_LU_ are pseudo-degenerate, respectively. This is because these orbitals consist of the fragment orbitals localized on the anthracene moieties. From Fig. 2, the frontier orbitals can be approximately represented as follows:

11 $${\psi }_{{\rm{LU}}}\approx \frac{1}{\sqrt{2}}({\varphi }_{{\rm{LU}}}(L)-{\varphi }_{{\rm{LU}}}(R)),\quad \quad {\psi }_{{\rm{NLU}}}\approx \frac{1}{\sqrt{2}}({\varphi }_{{\rm{LU}}}(L)+{\varphi }_{{\rm{LU}}}(R)),$$

12 $${\psi }_{{\rm{HO}}}\approx \frac{1}{\sqrt{2}}({\varphi }_{{\rm{HO}}}(L)+{\varphi }_{{\rm{HO}}}(R)),\quad \quad {\psi }_{{\rm{NHO}}}\approx \frac{1}{\sqrt{2}}({\varphi }_{{\rm{HO}}}(L)-{\varphi }_{{\rm{HO}}}(R)),$$

where *ϕ*_HO/LU_(*L*/*R*) denotes the fragment MOs consisting of the HOMO/LUMO of the anthracene moiety Left(L)/Right(R). These approximate expressions of the frontier orbitals will be used later.

**Figure 3:**
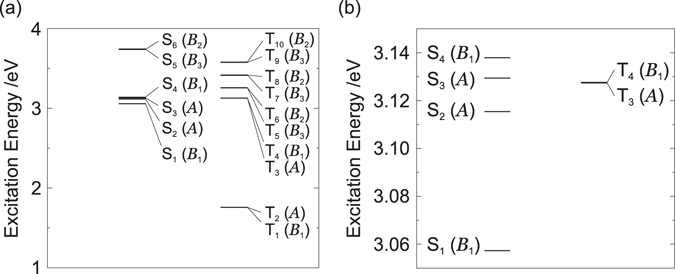
(**a**) Energy levels of the excited states in BD1 at the optimized structure for *S*_0_, and (**b**) an enlarged view of the relevant levels.

Franck–Condon (FC) excited states were calculated at the optimized structure for S_0_. Figure 3 shows the energy levels of the FC states. The singlet and triplet excited states from S_1_ to S_4_ and from T_1_ to T_4_ mainly consist of linear combinations of one-electron excited configurations of HOMO → LUMO, HOMO → NLUMO, NHOMO → LUMO, and NHOMO → NLUMO. The energy levels of T_3_ and T_4_ are close to those of S_1_, S_2_, S_3_, and S_4_. On the other hand, the energy levels of T_1_ and T_2_ are much lower than that of the lowest singlet excited state. Therefore, the TADF from the T_1_ and T_2_ states is not possible, as reported previously^18^.

In order to investigate roles of the higher excited states, the geometrical structures of T_4_, T_3_, S_4_, S_3_, S_2_, and S_1_ were optimized to discuss radiative and non-radiative transitions from the adiabatic excited states. Figures S1 and S2 in the Supplemental Material show the optimized structures of the relevant excited states. All the optimized structures show *D*_2_ symmetry. The T_4_ state has lower energy than T_3_ at the T_*n*_ optimized structure (*n* = 3, 4), as shown in Fig. S3 in Section S5 of the Supplemental Material. Hereafter, we will refer to the electronic state at a certain geometry corresponding to the T_*n*_ FC state as T_*n*_.

**Figure 4:**
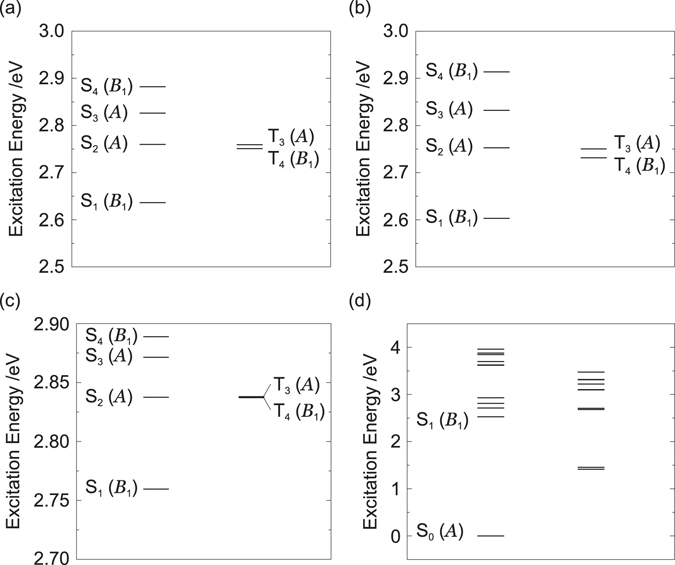
Energy levels of excited states at the optimized structures for (**a**) T_3_, (**b**) T_4_, (**c**) S_2_, and (**d**) S_1_.

Figures 4(a) and (b) show the energy levels of the excited states at the optimized structures for T_3_ and T_4_. Table 1 lists the triplet excited states at the optimized structure for T_3_. The selection rules are summarized in Section S6 of the Supplemental Material. Although T_3_ is close to S_2_ with ${\rm{\Delta }}{E}_{{T}_{3}-{S}_{2}}$ = 0.8 meV, RISC between T_3_ and S_2_ is symmetry forbidden (see Table S5). The electric dipole transition T_3_ → T_4_ and internal conversion (IC) T_3_ → T_4_ via *b*_1_ modes are allowed. Therefore, if the transition probability of T_3_ → T_4_ is large, a T_3_ exciton generated during electrical excitation is immediately converted into a T_4_ exciton. The transition rate will be discussed later by calculating off-diagonal VCCs.

**Table 1:** Triplet excited states at the optimized structure for T_3_.

	Excitation Energy		Major Configuration (CI Coefficient)
	eV	nm	
T_4_(*B*_1_)	2.7508	450.72	HO−1 → LU + 1(0.524),HO → LU(−0.455)
T_3_(*A*)	2.7591	449.37	HO−1 → LU(−0.501),HO → LU + 1(0.496)
T_2_(*A*)	1.4403	860.84	HO → LU + 1(0.502),HO−1 → LU(0.497)
T_1_(*B*_1_)	1.4252	869.93	HO → LU(−0.539),HO−1 → LU + 1(−0.458)

Table S7 of the Supplemental Material lists the triplet excited states at the optimized structure for T_4_. T_4_ is close to S_2_ with ${\rm{\Delta }}{E}_{{S}_{2}-{T}_{4}}$ = 21 meV, and RISC between T_4_ and S_2_ is symmetry allowed. Although the electric dipole transition T_4_ → T_1_ is symmetry forbidden, that between T_4_ and T_2_ is symmetry allowed. In addition, IC T_4_ → T_2_ with the help of *b*_1_ modes and IC T_4_ → T_1_ with the help of *α* modes are symmetry allowed. Only if the transitions of T_4_ → T_2_ and T_4_ → T_1_ are suppressed, a T_4_ exciton can be up-converted into an S_2_ exciton with thermal excitation. Otherwise, a T_4_ exciton is immediately relaxed into a T_1_ or T_2_ exciton, resulting in the decrease of the EQE. The validity of this up-conversion path will be confirmed later by calculating off-diagonal VCCs.

The exciton dynamics is also important after the up-conversion of a T_4_ exciton into a S_2_ exciton. Figure 4(c) and (d) show the energy levels of the excited states at the optimized structures for S_2_ and S_1_. The singlet states at the optimized structure for S_2_ are tabulated in Table S8 of the Supplemental Material. IC S_2_ → S_0_ via *a* modes and IC S_2_ → S_1_ via *b*_1_ modes are symmetry allowed. The electric dipole transition S_2_ → S_0_ is symmetry forbidden. On the other hand, the electric dipole transition S_2_ → S_1_ is symmetry allowed. Therefore, if the transition probability of IC S_2_ → S_0_ is small, an S_2_ exciton is relaxed into the S_1_ state. The singlet states at the optimized structure for S_1_ are listed in Table S9 of the Supplemental Material. S_1_ belongs to the *B*_1_ irreducible representation and is the fluorescent state, as indicated by the oscillator strengths *f* listed in Table S9 of the Supplemental Material.

**Figure 5:**
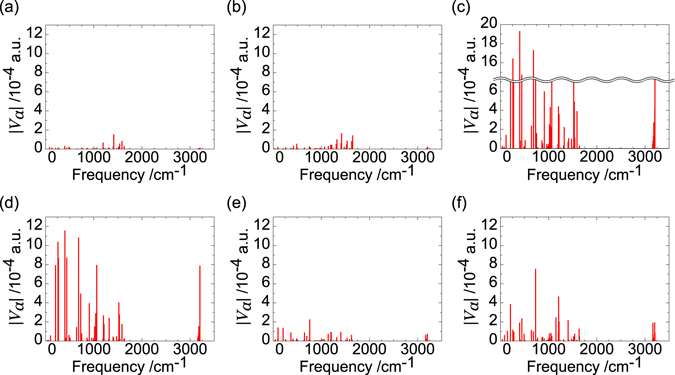
Off-diagonal VCCs of (**a**) T_4_@T_4_ → T_2_@T_4_, (**b**) T_4_@T_4_ → T_1_@T_4_, (**c**) T_3_@T_3_ → T_4_@T_3_, (**d**) S_2_@S_2_ → S_1_@S_2_, (**e**) S_2_@S_2_ → S_0_@S_2_, and (**f**) S_1_@S_1_ → S_0_@S_1_. @T_*n*_/@S_*n*_ denote the geometry used in optimization for the T_*n*_/S_*n*_ state.

As discussed above, if the transition probability of T_3_ → T_4_ is large, those of T_4_ → T_2_ and T_4_ → T_1_ are small, and that of S_2_ → S_0_ is small, then, the FvHT mechanism is possible: the high triplet excitons, T_3_ and T_4_, can be effectively converted into a fluorescent S_1_ exciton. The calculated off-diagonal VCCs are shown in Fig. 5. Vibrational modes with strong couplings are shown in Section S8 of the Supplemental Material. From Fig. 5, the transition probabilities of the ICs are in the following order:

13 $${{\rm{T}}}_{3}\to {{\rm{T}}}_{4} > {{\rm{S}}}_{2}\to {{\rm{S}}}_{1}\gg {{\rm{S}}}_{1}\to {{\rm{S}}}_{0}\gg {{\rm{S}}}_{2}\to {{\rm{S}}}_{0} > {{\rm{T}}}_{4}\to {{\rm{T}}}_{1} > {{\rm{T}}}_{4}\to {{\rm{T}}}_{2}\mathrm{.}$$

**Figure 6:**
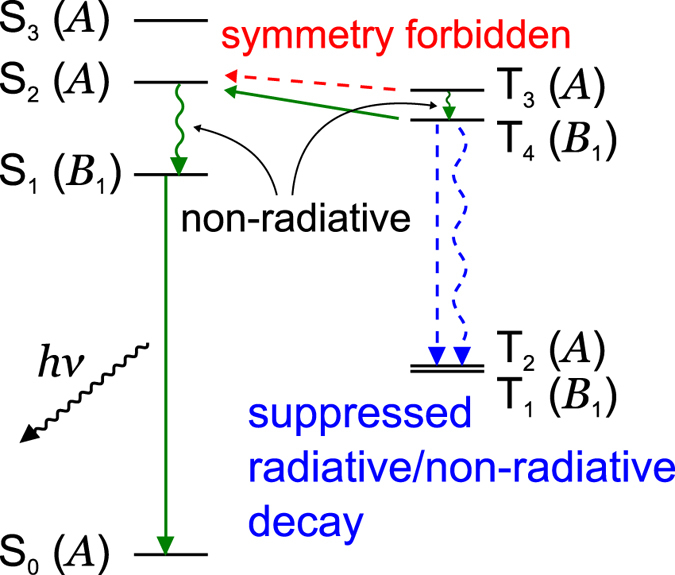
Scheme of the excited state dynamics in BD1. Horizontal solid arrows indicate RISC, vertical straight arrows indicate radiative transitions, and wavy arrows denote non-radiative transitions.

Based on the discussion of the adiabatic excited states and Eq. (13), the scheme of excited state dynamics is depicted in Fig. 6. From Fig. 5 and Eq. (13), the transition probabilities of these undesirable non-radiative processes for the present FvHT mechanism, S_2_ → S_0_, T_4_ → T_1_, and T_4_ → T_2_, are small. This is due to the disappearance of the overlap densities, as discussed later. It should be noted that the transition probabilities of the radiative transitions of S_2_ → S_0_, T_4_ → T_1_, and T_4_ → T_2_ are also small, because the TDMs as well as the VCCs depend on the overlap densities. On the other hand, the non-radiative transition probabilities of the ICs S_2_ → S_1_ and T_3_ → T_4_ are large. In addition, the transition probability of IC S_1_ → S_0_ is small enough for a S_1_ exciton to emit fluorescence. Accordingly, we can conclude that both T_3_ and T_4_ excitons generated during electrical excitation are effectively converted into a fluorescent S_1_ exciton.

## Discussion

**Figure 7:**
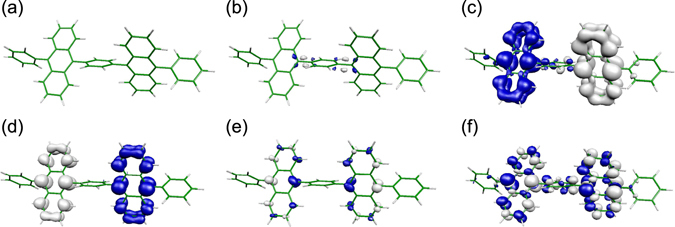
Overlap densities for (**a**) T_4_@T_4_–T_2_@T_4_, (**b**) T_4_@T_4_–T_1_@T_4_, (**c**) T_3_@T_3_–T_4_@T_3_, (**d**) S_2_@S_2_–S_1_@S_2_, (**e**) S_2_@S_2_–S_0_@S_2_, and (**f**) S_1_@S_1_–S_0_@S_1_, respectively. All isosurface values are the same, i.e. 1.0 × 10^−3^ a.u.

As was discussed in the previous section, the off-diagonal VCCs for T_3_–T_4_ and S_2_–S_1_ are large. On the other hand, the off-diagonal VCCs for T_4_–T_2_, T_4_–T_1_, and S_2_–S_0_ are small. These VCCs are the origin of the present FvHT mechanism. Figure 7 shows the overlap densities having the same isosurface values. Among them, the overlap densities for T_4_–T_2_, T_4_–T_1_, and S_2_–S_0_ disappear. This is the reason why the transitions of T_4_ → T_2_, T_4_ → T_1_, and S_2_ → S_0_ are suppressed (see also vibronic coupling density analyses for these transitions in Section S9 of the Supplemental Material). In this section, we discuss the disappearance mechanism of these overlap densities.

A TD-DFT wave function is given by

14 $${{\rm{\Psi }}}_{n}=\sum _{i\in {\rm{occ}}\,}\sum _{r\in {\rm{unocc}}}{C}_{i}^{r}{{\rm{\Phi }}}_{i}^{r},$$

where ${{\rm{\Phi }}}_{i}^{r}$ represents the electronic configuration of a single-electron excitation from occupied orbital *i* to vacant orbital *r*, and ${C}_{i}^{r}$ represents the CI coefficient. The overlap density between two excited states Ψ_*m*_ and Ψ_*n*_, *ρ* ^mn^, is given by

15 $${\rho }^{mn}=\sum _{i\in {\rm{occ}}}\sum _{\,r\in {\rm{unocc}}}\sum _{j\in {\rm{occ}}}\sum _{\,s\in {\rm{unocc}}}{D}_{j}^{s\ast }{C}_{i}^{r}\rho ({{\rm{\Phi }}}_{j}^{s},{{\rm{\Phi }}}_{i}^{r}),$$

where ${D}_{j}^{s}$ is the CI coefficient of another excited state *m*, and $\rho ({{\rm{\Phi }}}_{j}^{s},{{\rm{\Phi }}}_{i}^{r})$ denotes the overlap density between the two configurations.

Φ_0_ represents a ground state electron configuration, and its overlap density *ρ*_0_ is given by

16 $$\rho ({{\rm{\Phi }}}_{0},{{\rm{\Phi }}}_{0})=:{\rho }_{0}\mathrm{.}$$

The overlap densities for various configurations are summarized as follows,

17 $$\rho ({{\rm{\Phi }}}_{i}^{r},{{\rm{\Phi }}}_{i}^{r})={\rho }_{0}-{|{\psi }_{i}|}^{2}+{|{\psi }_{r}|}^{2},\quad \rho ({{\rm{\Phi }}}_{0},{{\rm{\Phi }}}_{i}^{r})={\psi }_{i}^{\ast }{\psi }_{r},\quad \rho ({{\rm{\Phi }}}_{i}^{r},{{\rm{\Phi }}}_{j}^{r})={\psi }_{i}^{\ast }{\psi }_{j}\quad (i\ne j),$$

18 $$\rho ({{\rm{\Phi }}}_{i}^{r},{{\rm{\Phi }}}_{i}^{s})={\psi }_{r}^{\ast }{\psi }_{s}\quad (r\ne s),\quad \rho ({{\rm{\Phi }}}_{i}^{r},{{\rm{\Phi }}}_{j}^{s})=0\quad (i\ne j,r\ne s),$$

where *ψ* represents a molecular orbital.

According to Tables 1, S7, S8, and S9, the approximate wave functions for the relevant excited states can be written as follows:

19 $${{\rm{\Psi }}}_{a}={C}_{1}^{a}{{\rm{\Phi }}}_{{\rm{HO}}}^{{\rm{LU}}}+{C}_{2}^{a}{{\rm{\Phi }}}_{{\rm{NHO}}}^{{\rm{NLU}}},\quad ({{\rm{T}}}_{4})$$

20 $${{\rm{\Psi }}}_{b}={C}_{3}^{b}{{\rm{\Phi }}}_{{\rm{HO}}}^{{\rm{NLU}}}+{C}_{4}^{b}{{\rm{\Phi }}}_{{\rm{NHO}}}^{{\rm{LU}}},\quad ({{\rm{S}}}_{2})$$

21 $${{\rm{\Psi }}}_{c}={C}_{1}^{c}{{\rm{\Phi }}}_{{\rm{HO}}}^{{\rm{LU}}}+{C}_{2}^{c}{{\rm{\Phi }}}_{{\rm{NHO}}}^{{\rm{NLU}}},\quad ({{\rm{T}}}_{1})$$

22 $${{\rm{\Psi }}}_{d}={{\rm{\Phi }}}_{{\rm{HO}}}^{{\rm{LU}}},\quad ({{\rm{S}}}_{1})$$

23 $${{\rm{\Psi }}}_{e}={C}_{3}^{e}{{\rm{\Phi }}}_{{\rm{HO}}}^{{\rm{NLU}}}+{C}_{4}^{e}{{\rm{\Phi }}}_{{\rm{NHO}}}^{{\rm{LU}}},\quad ({{\rm{T}}}_{2})$$

24 $${{\rm{\Psi }}}_{0}={{\rm{\Phi }}}_{0},\quad ({{\rm{S}}}_{0})$$

where the set of the CI coefficients is assumed to satisfy the following relation:

25 $${C}_{1}^{a}\approx -{C}_{2}^{a}\approx {C}_{3}^{b}\approx -{C}_{4}^{b}\approx {C}_{1}^{c}\approx {C}_{2}^{c}\approx {C}_{3}^{e}\approx {C}_{4}^{e}\approx c\mathrm{.}$$

In addition, we should consider the conditions of orbital overlap densities among the frontier orbitals (see Eqs (11) and (12)). Since *ϕ*_*i*_(*L*) and *ϕ*_*j*_(*R*) are the localized fragment MOs, *ϕ*_*i*_(*L*)*ϕ*_*j*_(*R*) ≈ 0 (*i*, *j* = HO, NHO, LU, and NLU). Accordingly,

26 $${|{{\psi }}_{{\rm{LU}}}|}^{2}\approx {|{{\psi }}_{{\rm{NLU}}}|}^{2},\quad {{\psi }}_{{\rm{HO}}}{{\psi }}_{{\rm{NLU}}}\approx {{\psi }}_{{\rm{NHO}}}{{\psi }}_{{\rm{LU}}},\quad {|{{\psi }}_{{\rm{HO}}}|}^{2}\approx {|{{\psi }}_{{\rm{NHO}}}|}^{2},\quad {{\psi }}_{{\rm{HO}}}{{\psi }}_{{\rm{LU}}}\approx {{\psi }}_{{\rm{NHO}}}{{\psi }}_{{\rm{NLU}}},$$

27 $${{\psi }}_{{\rm{HO}}}{{\psi }}_{{\rm{NHO}}}\ne {{\psi }}_{{\rm{LU}}}{{\psi }}_{{\rm{NLU}}}.$$

In general, these conditions can be satisfied in systems with pseudo-degenerate electronic states.

In order to elucidate the reason for the disappearance of the overlap densities shown in Fig. 7, we discuss the overlap densities between approximate excited wave functions.

(Case 1: Ψ_a_ and Ψ_e_) This case corresponds to T_4_–T_2_. The overlap density between Ψ_*a*_ and Ψ_*e*_ is given by

28 $${\rho }^{ae}=({C}_{1}^{a}{C}_{3}^{e}+{C}_{2}^{a}{C}_{4}^{e}){{\psi }}_{{\rm{LU}}}{{\psi }}_{{\rm{NLU}}}+({C}_{1}^{a}{C}_{4}^{e}+{C}_{2}^{a}{C}_{3}^{e}){{\psi }}_{{\rm{HO}}}{{\psi }}_{{\rm{NHO}}}\mathrm{.}$$

From the condition for the CI coefficients, (Eq. 25),

29 $${C}_{1}^{a}{C}_{3}^{e}+{C}_{2}^{a}{C}_{4}^{e}\approx \mathrm{0,}\quad {C}_{1}^{a}{C}_{4}^{e}+{C}_{2}^{a}{C}_{3}^{e}\approx 0.$$

Thus, the overlap density between Ψ_*a*_ and Ψ_*e*_ is cancelled out. Note that the disappearance of overlap density originates from the condition of the CI coefficients.

(Case 2: Ψ_*a*_ and Ψ_*c*_) This case corresponds to T_4_–T_1_. The overlap density between Ψ_*a*_ and Ψ_*c*_ is given by

30 $${\rho }^{ac}={C}_{1}^{a}{C}_{1}^{c}({\rho }_{0}-{|{{\psi }}_{{\rm{HO}}}|}^{2}+{|{{\psi }}_{{\rm{LU}}}|}^{2})+{C}_{2}^{a}{C}_{2}^{c}({\rho }_{0}-{|{{\psi }}_{{\rm{NHO}}}|}^{2}+{|{{\psi }}_{{\rm{NLU}}}|}^{2})\mathrm{.}$$

From the condition for the CI coefficients,

31 $${\rho }^{ac}\approx {c}^{2}({|{{\psi }}_{{\rm{NHO}}}|}^{2}-{|{{\psi }}_{{\rm{HO}}}|}^{2}+{|{{\psi }}_{{\rm{LU}}}|}^{2}-{|{{\psi }}_{{\rm{NLU}}}|}^{2})\mathrm{.}$$

From the condition for the orbital densities, Eq. (26),

32 $${\rho }^{ac}\approx 0.$$

Note that the disappearance of overlap density originates from the condition of the orbital densities of the frontier orbitals as well as that of the CI coefficients.

(Case 3: Ψ_*b*_ and Ψ_0_) This case corresponds to S_2_–S_0_. The overlap density between Ψ_*b*_ and Ψ_0_ is given by

33 $${\rho }^{b0}\approx c({{\psi }}_{{\rm{HO}}}{{\psi }}_{{\rm{NLU}}}-{{\psi }}_{{\rm{NHO}}}{{\psi }}_{{\rm{LU}}}\mathrm{).}$$

According to Eq. (26), *ρ* ^*b*0^ is cancelled out (see also Fig. S16 in the Supplemental Material). Note that the disappearance of overlap density originates from the condition of the orbital overlap densities of the frontier orbitals as well as that of the CI coefficients.

(Case 4: Ψ_*b*_ and Ψ_*d*_) This case corresponds to S_2_–S_1_. According to Eq. (26), the overlap density between Ψ_*b*_ and Ψ_*d*_ is given by

34 $${\rho }^{bd}\approx c({{\psi }}_{{\rm{NLU}}}{{\psi }}_{{\rm{LU}}}-{{\psi }}_{{\rm{NHO}}}{{\psi }}_{{\rm{HO}}})\ne 0.$$

Thus, *ρ* ^*bd*^ is not cancelled out.

General cases are discussed in Section S11 of the Supplemental Material. The reduced overlap densities of Cases 1–3, which originate from the pseudo degeneracy, are responsible for the suppression of undesirable radiative and non-radiative transitions, T_3_ → T_2_, T_3_ → T_1_, and S_2_ → S_0_, for the FvHT mechanism.

In conclusion, we proposed a *fluorescence via higher triplets* (*FvHT*) emitting mechanism for OLEDs based on a bisanthracene derivative, BD1. This mechanism is valid as long as all transitions from T_*m*_ (*m* > 1) to all lower T_*n*_ (*m* > *n* ≥ 1) are suppressed. In BD1, we found that this condition is satisfied because of its pseudo-degenerate electronic structure. The undesirable radiative and non-radiative transitions can be suppressed by utilizing the pseudo-degeneracy. We also discussed general conditions for the suppression of radiative and non-radiative transitions in a pseudo-degenerate system. Recently, Hu *et al*. have reviewed the RISC from upper triplet levels to excited singlet^19^. Since some molecules are expected to have pseudo-degenerate electronic structures, the RISCs of such systems might be elucidated from the point of view of the present mechanism. The general conditions are applicable not only for transitions in a molecule, but also for exciton migrations in the solid phase. The design principle based on the FvHT mechanism allows the use of asymmetric molecules, differently from iST and SC-TADF based on the selection rules for a molecular symmetry group. Finally, we propose a superordinate concept, *fluorescence via RISC* (*FvRISC*) from T_1_ or higher triplet states. This concept includes TADF, SC-TADF, iST, and FvHT. The concept of FvRISC enables us to overcome the singlet exciton formation ratio of 25% for electrical excitations and to realize highly efficient OLEDs with a wide variety of molecular structures and symmetries.

## Methods

The optimized structure of BD1 in the ground state was obtained. The structure was confirmed to be the minimum energy structure using vibrational analysis. In order to discuss radiative and non-radiative transition processes from excited states, excited adiabatic (AD) states were obtained by carrying out geometry optimizations in the excited states. The normal modes were also obtained by vibrational analyses. The vibrational analyses were carried out for S_0_ at the optimized structures for the excited states. These calculations were performed at the B3LYP/6-311 + G(d,p) and TD-B3LYP/6-311 + G(d,p) levels of theory for the ground and excited states, respectively. In the excited state calculations, ten singlet and ten triplet states were taken into consideration. Off-diagonal VCCs between triplet states T_*m*_–T_*n*_, as well as singlet states S_*m*_–S_*n*_, which govern the non-radiative transition rates, were calculated at the AD states. Furthermore, in order to analyze the results of the VCC calculations, VCD analyses were carried out for strong coupling modes. The electronic and vibrational states were calculated using Gaussian 09 Revision D.01^20^, while the VCC calculations and VCD analyses were performed using our in-house codes.

## Electronic supplementary material

Supplemental Material

## References

[1] Adachi C (2014). Third-generation organic electroluminescence materials. Jpn. J. Appl. Phys..

[2] Endo A (2009). Thermally activated delayed fluorescence from Sn^4+^-porphyrin complexes and their application to organic light emitting diodes – a novel mechanism for electroluminescence. Adv. Mater..

[3] Tanaka H, Shizu K, Miyazaki H, Adachi C (2012). Efficient green thermally activated delayed fluorescence (TADF) from a phenoxazine-triphenyltriazine (PXZ-TRZ) derivative. Chem. Commun..

[4] Uoyama H, Goushi K, Shizu K, Nomura H, Adachi C (2012). Highly efficient organic light-emitting diodes from delayed fluorescence. Nature.

[5] Lee SY, Yasuda T, Nomura H, Adachi C (2012). High-efficiency organic light-emitting diodes utilizing thermally activated delayed fluorescence from triazine-based donor-acceptor hybrid molecules. Appl. Phys. Lett..

[6] Nakagawa T, Ku S-Y, Wong K-T, Adachi C (2012). Electroluminescence based on thermally activated delayed fluorescence generated by a spirobifluorene donor-acceptor structure. Chem. Commun..

[7] Méhes G, Nomura H, Zhang Q, Nakagawa T, Adachi C (2012). Enhanced electroluminescence efficiency in a spiro-acridine derivative through thermally activated delayed fluorescence. Angew. Chem., Int. Ed..

[8] Kaji H (2015). Purely organic electroluminescent material realizing 100% conversion from electricity to light. Nat. Commun..

[9] Sato T (2013). Vibronic coupling density and related concepts. J. Phys.: Conf. Ser..

[10] Uejima M, Sato T, Yokoyama D, Tanaka K, Park J-W (2014). Quantum yield in blue-emitting anthracene derivatives: vibronic coupling density and transition dipole moment density. Phys. Chem. Chem. Phys..

[11] Sato T, Uejima M, Tanaka K, Kaji H, Adachi C (2015). A light-emitting mechanism for organic light-emitting diodes: molecular design for inverted singlet-triplet structure and symmetry-controlled thermally activated delayed fluorescence. J. Mater. Chem. C.

[12] Yao L (2014). Highly efficient near-infrared organic light-emitting diode based on a butterfly-shaped donor-acceptor chromophore with strong solid-state fluorescence and a large proportion of radiative excitons. Angew. Chem., Int. Ed..

[13] Sato T (2015). Fluorescence via reverse intersystem crossing from higher triplet states. J. Comput. Chem., Jpn.

[14] Longuet-Higgins HC (1956). The Electronic States of Composite Systems. Proc. R. Soc. London, Ser. A.

[15] Hayashi M (1998). Ab initio calculations of radiationless transitions between excited and ground singlet electronic states of ethylene. J. Chem. Phys..

[16] Niu Y (2010). Theory of Excited State Decays and Optical Spectra: Application to Polyatomic Molecules. J. Phys. Chem. A.

[17] Fischer, G. *Vibronic Coupling: The Interaction Between the Electronic and Nuclear Motions*. Academic Press, London (1984).

[18] Hu J-Y (2014). Bisanthracene-based donor-acceptor-type light-emitting dopants: Highly efficient deep-blue emission in organic light-emitting devices. Adv. Funct. Mater..

[19] Hu D (2015). Reverse intersystem crossing from upper triplet levels to excited singlet: a ‘hot excition’ path for organic light-emitting diodes. Phil. Trans. R. Soc. A.

[20] Frisch, M. J. *et al*. Gaussian 09 Revision D.01 http://gaussian.com/ (Gaussian Inc., Wallingford CT 2009.)

