# Supplementary material for: Fluorescence via Reverse Intersystem Crossing from Higher Triplet States in a Bisanthracene Derivative
Source: Sci Rep. 2017 Jul 6;7:4820. doi: 10.1038/s41598-017-05007-7 (PMC5500583; doi:10.1038/s41598-017-05007-7)
Supplement: Supplementary file 1 — Supplemental Material [file 41598_2017_5007_MOESM1_ESM.pdf]

Supplemental Material

Fluorescence via Reverse Intersystem Crossing from Higher Triplet States in a Bisanthracene Derivative

Tohru Sato,[*a,b] **Rika Hayashi**[c], **Naoki Haruta**[a], **and Yong-Jin Pu**[d,e]

December 20, 2016

[a] *Department of Molecular Engineering, Graduate School of Engineering, Kyoto University, Nishikyo-ku, Kyoto 615-8510, Japan; Tel: +81-75-383-2544; Fax: +81-75-383-2799; E-mail: tsato@moleng.kyoto-u.ac.jp*

[b] *Unit of Elements Strategy Initiative for Catalysts & Batteries, Kyoto University, Nishikyo-ku, Kyoto 615-8510, Japan*

[c] *Undergraduate School of Industrial Chemistry, Faculty of Engineering, Kyoto University, Nishikyo-ku, Kyoto 615-8510, Japan*

[d] *Department of Organic Device Engineering and Research Center for Organic Electronics, Yamagata University, 4-3-16, Johnan, Yonezawa, 992-8510, Japan*

[e] *PRESTO (Sakigake), JST*

Contents

S1 Radiative and Non-Radiative Transition Rates S3

S2 Vibronic Coupling Density and Transition Dipole Moment Density S4

S3 Optimized Structures of BD1 in Excited States S5

S4 Imposition of D_{2h}-Symmetry Constraint on BD1 . . . . S6

S4.1 Singlet and Triplet Excited States S6

S4.2 Subduction from D_{2h} to D_2 . . . . S6

S5 Level Shifts of Triplet Excites States during Optimizations S7

S6 Selection Rules S8

S7 CI Coefficients of the Triplet States S11

S8 Vibrational Modes with Strong Couplings S12

S9 Vibronic Coupling Density Analyses S16

S10 Orbital Overlap Densities S19

S11 General Conditions for Disappearances of Overlap Densities in Pseudo-Degenerate Systems . S20

S1 Radiative and Non-Radiative Transition Rates

We consider transition from the initial vibronic state $|\Phi_{mi}\rangle = |\Psi_m\rangle |\chi_i\rangle$ to the final one $|\Phi_{nj}\rangle = |\Psi_n\rangle |\chi_j\rangle$, where $|\Psi_m\rangle$ and $|\chi_i\rangle$ denote the initial electronic and vibrational states, and $|\Psi_n\rangle$ and $|\chi_j\rangle$ stand for the final electronic and vibrational states, respectively. Radiative and non-radiative transition rate constants between these vibronic states can be obtained as per the Fermi's golden rule, which describes a transition rate constant between quantum states in a general manner, as described in our previous papers[1,2]. Summing radiative transition rate constants over all the vibrational states, the radiative transition rate constant k_r from the initial electronic state $|\Psi_m\rangle$ to the final one $|\Psi_n\rangle$ is given by

$$k_r = \int_0^\infty d\omega \frac{4\omega^3}{3c^3} \sum_{i,j} P_{mi}(T) |\boldsymbol{\mu}_{mn}|^2 |\langle \chi_{mi} | \chi_{nj} \rangle|^2 \delta(\hbar\omega - E_{mi} + E_{nj}), \tag{S1}$$

where ω denotes an angular frequency of an emitted photon, c is the speed of light, $P_{mi}(T)$ stands for the statistical weight of $|\Phi_{mi}\rangle$ at the temperature T, $\boldsymbol{\mu}_{nm}$ is the transition dipole moment between $|\Psi_m\rangle$ and $|\Psi_n\rangle$, and $\hbar$ is the reduced Planck Constant. On the other hand, the non-radiative transition rate constant $k_{nr,\alpha}$ via mode α is given by

$$k_{nr,\alpha} = \frac{2\pi}{\hbar} \sum_{i,j} P_{mi}(T) |V_{mn,\alpha}|^2 |\langle \chi_{mi} | Q_\alpha | \chi_{nj} \rangle|^2 \delta(E_{mi} - E_{nj}), \tag{S2}$$

where V_α^{mn} is the off-diagonal vibronic coupling constant between $|\Psi_m\rangle$ and $|\Psi_n\rangle$ with respect to mode α, and Q_α denotes a mass-weighted normal coordinate of mode α.

S2 Vibronic Coupling Density and Transition Dipole Moment Density

Suppose that an operator $\hat{O}$ consists of one-electron operators $\hat{o}$ without any differential operators,

$$\hat{O} = \sum_{i=1}^{N} \hat{o}(\boldsymbol{r}_i), \tag{S3}$$

where $\boldsymbol{r}_i$ denotes the spatial coordinate of electron i. A matrix element of $\hat{O}$ is given by[1]

$$\begin{aligned} O_{mn} &= \int \cdots \int \Psi_m^* \hat{O} \Psi_n d^4\boldsymbol{x}_1 \cdots d^4\boldsymbol{x}_N \\ &= \sum_{i=1}^{N} \int \left[\int \cdots \int \Psi_m{}^* \Psi_n d^4\boldsymbol{x}_1 \cdots d^4\boldsymbol{x}_{i-1} ds_i d^4\boldsymbol{x}_{i+1} \cdots d^4\boldsymbol{x}_N \right] \hat{o}(\boldsymbol{r}_i) d^3\boldsymbol{r}_i \\ &= \sum_{i=1}^{N} \int \left[\frac{1}{N} \rho^{mn}(\boldsymbol{r}_i) \right] \hat{o}(\boldsymbol{r}_i) d^3\boldsymbol{r}_i \\ &= \int \rho^{mn}(\boldsymbol{r}) \hat{o}(\boldsymbol{r}) d^3\boldsymbol{r}, \end{aligned} \tag{S4}$$

where $\boldsymbol{x}_i = (\boldsymbol{r}_i, s_i)$ with spatial coordinate $\boldsymbol{r}_i$ and spin coordinate s_i for electron i. $\rho^{mn}(\boldsymbol{r}) \times \hat{o}(\boldsymbol{r})$ is a density form of the matrix element O_{mn}. It should be noted that any approximation is not employed in this derivation.

An electric dipole moment operator $\hat{\boldsymbol{\mu}}$ is an example of $\hat{O}$:

$$\hat{\boldsymbol{\mu}} := \sum_{i=1}^{N} -e\boldsymbol{r}_i. \tag{S5}$$

Therefore, a transition dipole moment $\boldsymbol{\mu}_{mn}$ between electronic states m and n is given by the integral of a transition dipole moment density $\boldsymbol{\tau}_{mn}(\boldsymbol{r}) = \rho^{mn}(\boldsymbol{r}) \times (-e\boldsymbol{r})$[1,2].

An off-diagonal vibronic coupling constant V_α^{mn} is

$$\begin{aligned} V_\alpha^{mn} &= \int \cdots \int \Psi_m^* \left(\frac{\partial U_{ne}}{\partial Q_\alpha} \right)_{\boldsymbol{R}_0} \Psi_n d^4\boldsymbol{x}_1 \cdots d^4\boldsymbol{x}_N + \int \cdots \int \Psi_m^* \left(\frac{\partial U_{nn}}{\partial Q_\alpha} \right)_{\boldsymbol{R}_0} \Psi_n d^4\boldsymbol{x}_1 \cdots d^4\boldsymbol{x}_N \\ &= \int \cdots \int \Psi_m^* \left(\frac{\partial U_{ne}}{\partial Q_\alpha} \right)_{\boldsymbol{R}_0} \Psi_n d^4\boldsymbol{x}_1 \cdots d^4\boldsymbol{x}_N, \end{aligned} \tag{S6}$$

where U_{ne} denotes the sum of nuclear-electronic potentials, U_{nn} stands for the sum of nuclear-nuclear potentials, and Q_α is a mass-weighted normal coordinate of mode α. Since $(\partial U_{ne}/\partial Q_\alpha)_{\boldsymbol{R}_0}$ can be written as the sum of one-electron potential derivatives:

$$\left(\frac{\partial U_{ne}}{\partial Q_\alpha} \right)_{\boldsymbol{R}_0} = \sum_{i=1}^{N} v_\alpha(\boldsymbol{r}_i), \tag{S7}$$

V_α^{mn} is given by the integral of a vibronic coupling density $\eta_\alpha^{mn}(\boldsymbol{r}) = \rho^{mn}(\boldsymbol{r}) \times v_\alpha(\boldsymbol{r})$[1,2].

S3 Optimized Structures of BD1 in Excited States

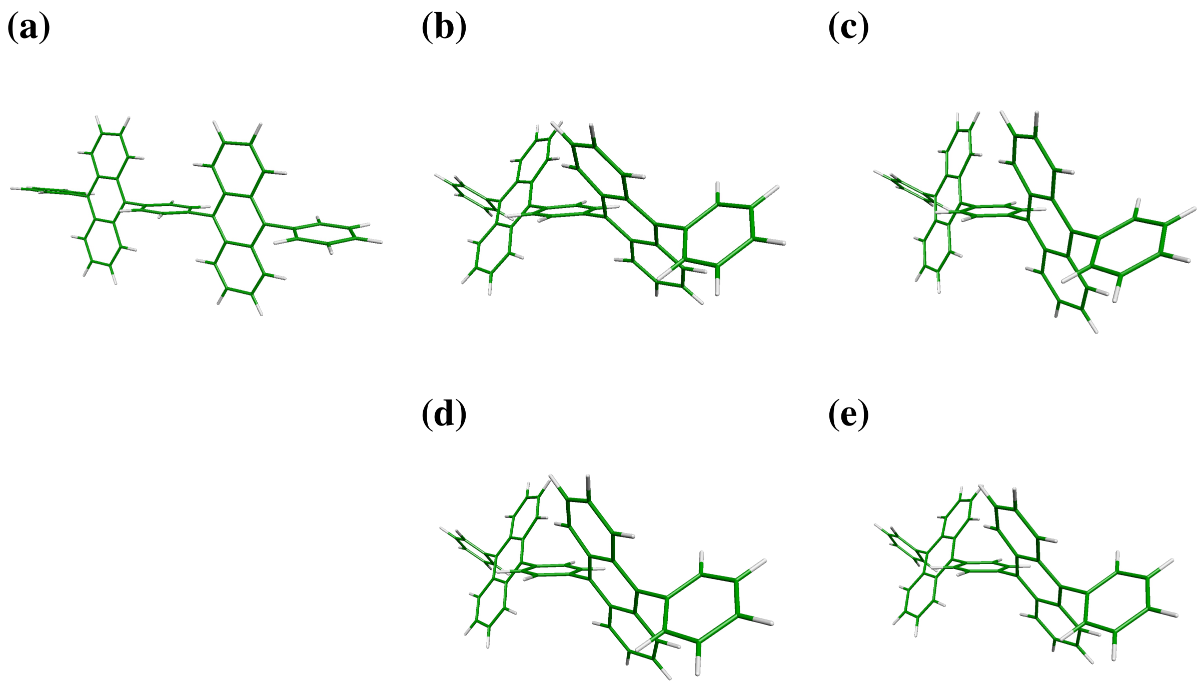

Figure S1: Optimized structures of BD1 for (a) S_0, (b) S_1, (c) S_2, (d) T_3, and (e) T_4.

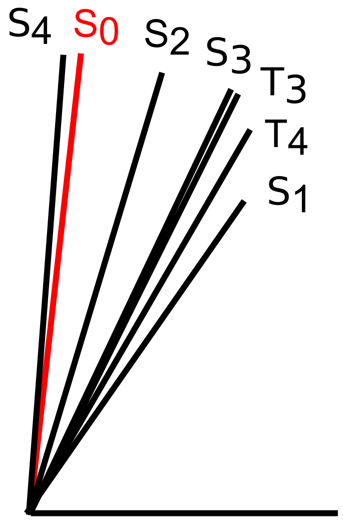

Figure S2: Dihedral angles (C1–C2–C3–C4) between the anthracene and benzene moieties for the geometry-optimized excited states.

S4 Imposition of D_{2h}-Symmetry Constraint on BD1

S4.1 Singlet and Triplet Excited States

Table S1: Excited states of BD1 at the optimized structure for S_0 with a D_{2h}-symmetry constraint.

State		Excitation Energy		Oscillator Strength
		eV	nm	
T_1	$^3B_{3u}$	2.2072	561.72	0.0000
T_2	^3A_g	2.2078	561.57	0.0000
S_1	$^1B_{3u}$	3.5520	349.05	0.5269
S_2	^1A_g	3.6186	342.63	0.0000
T_3	$^3B_{1g}$	3.7529	330.37	0.0000
T_4	$^3B_{2u}$	3.7533	330.34	0.0000

S4.2 Subduction from D_{2h} to D_2

$$A_g \downarrow D_2 = A \tag{S8}$$

$$B_{1g} \downarrow D_2 = B_1 \tag{S9}$$

$$B_{2g} \downarrow D_2 = B_2 \tag{S10}$$

$$B_{3g} \downarrow D_2 = B_3 \tag{S11}$$

$$A_u \downarrow D_2 = A \tag{S12}$$

$$B_{1u} \downarrow D_2 = B_1 \tag{S13}$$

$$B_{2u} \downarrow D_2 = B_2 \tag{S14}$$

$$B_{3u} \downarrow D_2 = B_3 \tag{S15}$$

S5 Level Shifts of Triplet Excites States during Optimizations

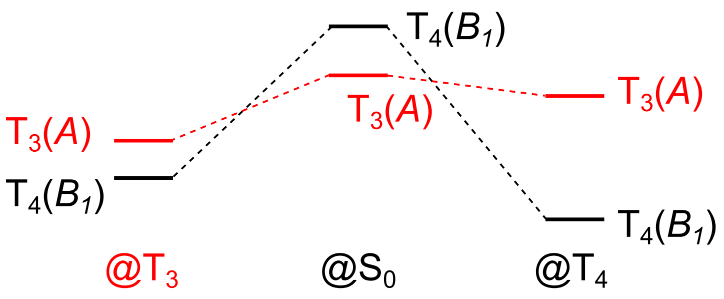

Figure S3: Level shifts of triplet excited states of BD1 during geometry optimizations.

S6 Selection Rules

Since all the optimized structures show D_2 symmetry, the selection rules for transition dipole moment, spin-orbit coupling, and vibronic coupling within D_2 symmetry are discussed here. Table S2 is the character table of the D_2 point group. The direct products of the irreducible representations (irreps) are tabulated in Table S3. From Table S1, the components of the electric dipole operator, $\hat{\mu}_x$, $\hat{\mu}_y$, $\hat{\mu}_z$ transform according to the B_3, B_2, and B_1 irreps, respectively, and the components of the orbital angular momentum operator, $\hat{L}_x$, $\hat{L}_y$, $\hat{L}_z$ transform according to the B_3, B_2, and B_1 irreps, respectively. Based on Table S3, the selection rules for transition dipole moment, spin-orbit coupling, and vibronic coupling were obtained and are listed in Tables S4, S5, and S6. According to Tables S5 and S6, an electric dipole transition or intersystem crossing between electronic states with the same irrep is symmetry forbidden.

Table S6 lists the selection rule for vibronic coupling with D_2 symmetry. The vibrational degrees of freedom in BD1 are decomposed into irreps as follows:

$$\Gamma_{vib} = 54a_1 \oplus 52b_1 \oplus 58b_2 \oplus 58b_3. \tag{S16}$$

According to Table S6, the number of vibronically active modes accounts for 1/4 of all the modes for every non-radiative transition $m \to n$. For example, in non-radiative transition $S_1 \to S_0$, the vibronically active modes are fifty two b_1 modes because the S_1 state transforms as B_1.

Table S2: Character table of D_2.

$\boldsymbol{D_2}$	E	$C_2(z)$	$C_2(y)$	$C_2(x)$	$h=4$	
A	1	1	1	1		x^2,y^2,z^2
B_1	1	1	-1	-1	z,R_z	xy
B_2	1	-1	1	-1	y,R_y	zx
B_3	1	-1	-1	1	x,R_x	yz

Table S3: Direct products of irreducible representations in D_2.

$\boldsymbol{D_2}$	A	B_1	B_2	B_3
A	A	B_1	B_2	B_3
B_1		A	B_3	B_2
B_2			A	B_1
B_3				A

Table S4: Selection rule of the transition dipole moment in D_2. $-$ denotes a forbidden transition, and μ_i $(i = x, y, z)$ denotes a symmetry-allowed component of transition dipole moment $\langle m|\hat{\boldsymbol{\mu}}|n\rangle$.

$m/\,n$	A	B_1	B_2	B_3
A	$-$	μ_z	μ_y	μ_x
B_1		$-$	μ_x	μ_y
B_2			$-$	μ_z
B_3				$-$

Table S5: Selection rule of spin-orbit coupling in D_2. $-$ denotes a forbidden transition, and L_i $(i = x, y, z)$ denotes a symmetry-allowed component of orbital angular moment $\langle m|\hat{\boldsymbol{L}}|n\rangle$.

$m/\,n$	A	B_1	B_2	B_3
A	$-$	L_z	L_y	L_x
B_1		$-$	L_x	L_y
B_2			$-$	L_z
B_3				$-$

Table S6: Selection rule of vibronic coupling in D_2. $-$ denotes a forbidden transition, and V_Γ ($\Gamma = A, B_1, B_2, B_3$) denotes a symmetry-allowed component of vibronic coupling constant $\langle m|\hat{V}_\Gamma|n\rangle$, where $\hat{V}_\Gamma = (\partial H/\partial Q_\Gamma)$ with normal coordinate Q_Γ.

m/ n	A	B_1	B_2	B_3
A	V_{a_1}	V_{b_1}	V_{b_2}	V_{b_3}
B_1		V_{a_1}	V_{b_3}	V_{b_2}
B_2			V_{a_1}	V_{b_1}
B_3				V_{a_1}

S7 CI Coefficients of the Triplet States

Table S7: Triplet excited states at the optimized structure for T_4.

	Excitation Energy eV	nm	Major Configuration (CI coefficient)
$T_3(A)$	2.7503	450.81	HO-1→LU(-0.501),HO→LU+1(0.495)
$T_4(B_1)$	2.7314	453.93	HO-1→LU+1(0.522),HO→LU(-0.436)
$T_2(A)$	1.4590	849.77	HO→LU+1(0.501),HO-1→LU(0.495)
$T_1(B_1)$	1.4345	864.29	HO→LU(-0.549),HO-1→LU+1(-0.444)

Table S8: Singlet excited states at the optimized structure for S_2.

	Excitation Energy eV	nm	Osc.	Major configuration (CI coefficient)
$S_4(B_1)$	2.8889	429.18	0.1041	HO-1→LU+1(0.698)
$S_3(A)$	2.8714	431.80	0.0000	HO-1→LU(0.591),HO→LU+1(0.379)
$S_2(A)$	2.8375	436.95	0.0000	HO→LU+1(0.593),HO-1→LU(-0.382)
$S_1(B_1)$	2.7596	449.28	0.3131	HO→LU(0.698)

Table S9: Singlet excited states at the optimized structure for S_1.

	Excitation Energy eV	nm	Osc.	Major configuration (CI coefficient)
$S_4(B_1)$	2.9295	423.23	0.0981	HO-1→LU+1(0.704)
$S_3(A)$	2.8099	441.24	0.0000	HO-1→LU(-0.546),HO→LU+1(-0.442)
$S_2(A)$	2.7124	457.09	0.0000	HO→LU+1(0.547),HO-1→LU(-0.444)
$S_1(B_1)$	2.5267	490.70	0.4800	HO→LU(-0.705)

S8 Vibrational Modes with Strong Couplings

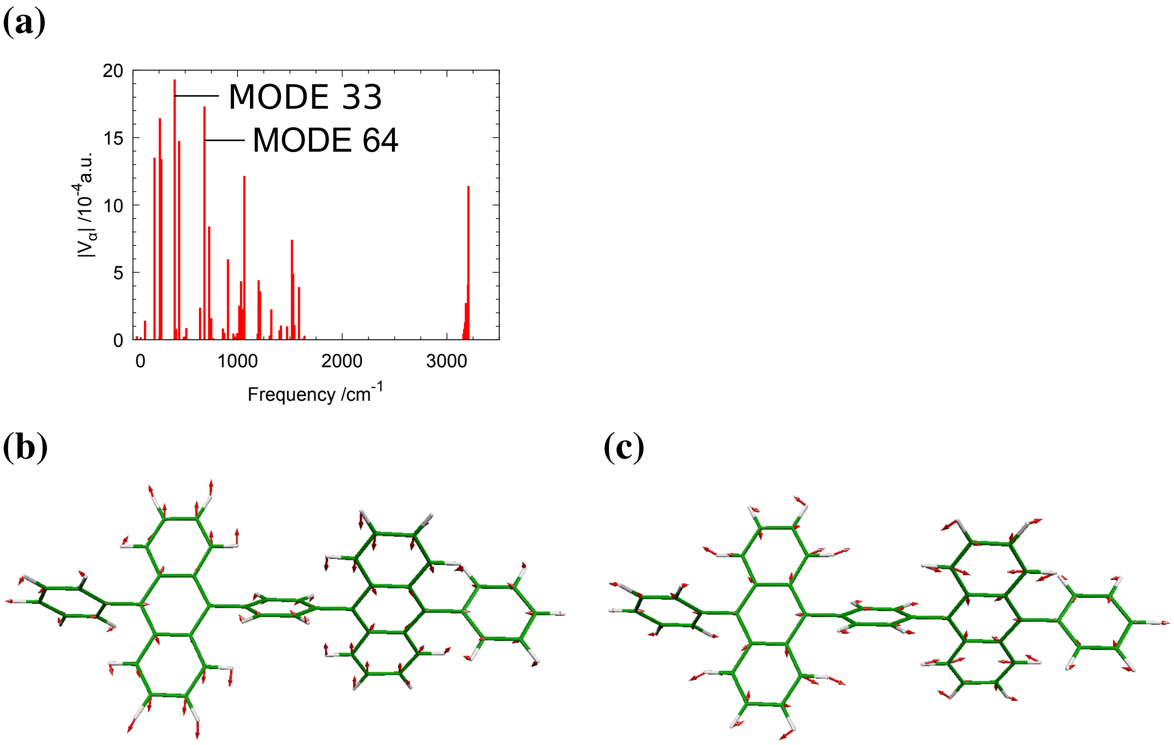

Figure S4: (a) Off-diagonal VCCs between T_3 and T_4 at the T_3 optimized structure, and strong coupling modes: (b) mode 33 and (c) mode 64.

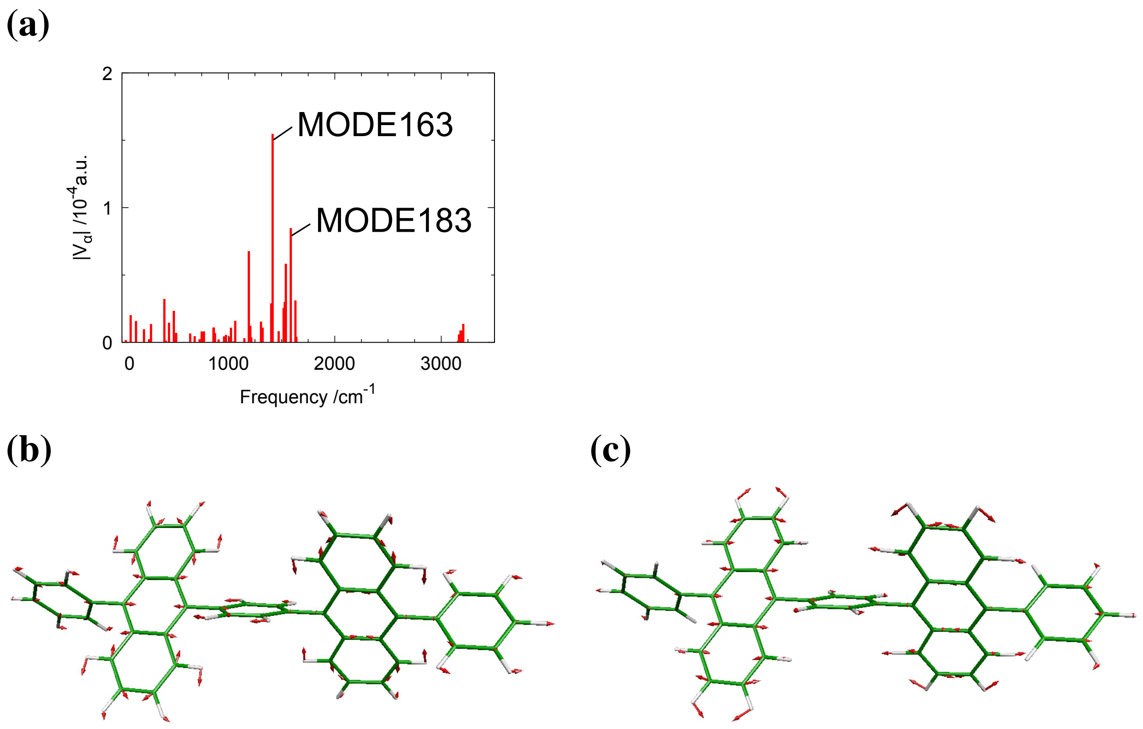

Figure S5: (a) Off-diagonal VCCs between T_4 and T_2 at the T_4 optimized structure, and strong coupling modes: (b) mode 163 and (c) mode 183.

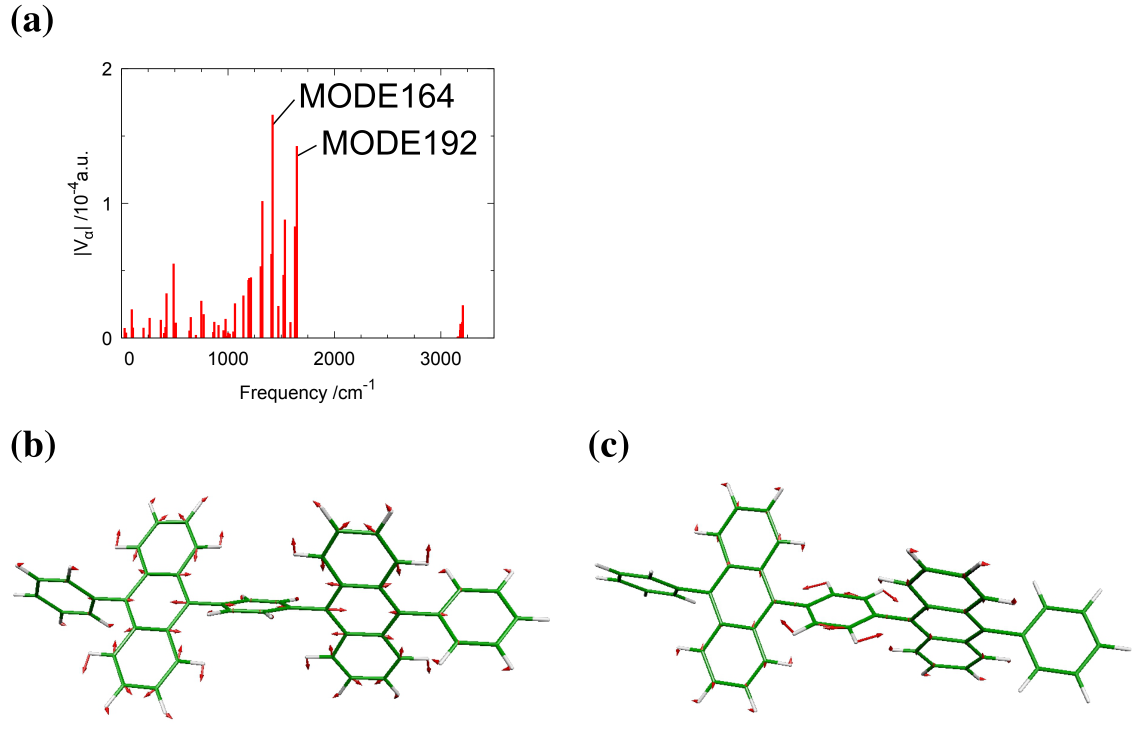

Figure S6: (a) Off-diagonal VCCs between T_4 and T_1 at the T_4 optimized structure, and strong coupling modes: (b) mode 164 and (c) mode 192.

(a)

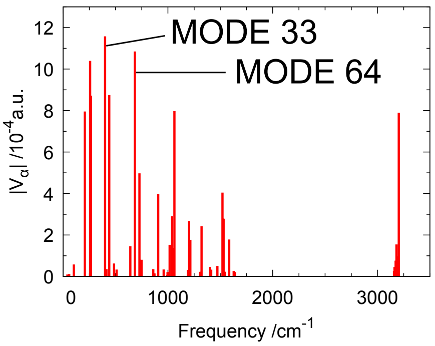

(b) **(c)**

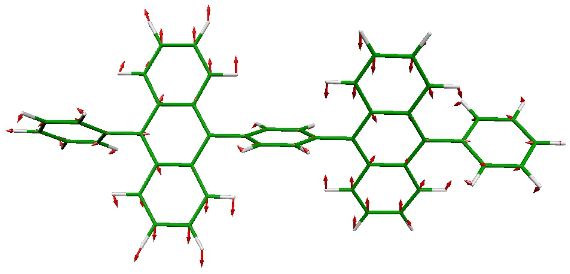

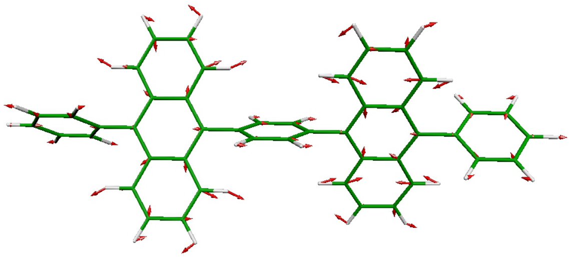

Figure S7: (a) Off-diagonal VCCs between S_2 and S_1 at the S_2 optimized structure, and strong coupling modes: (b) mode 33 and (c) mode 64.

(a)

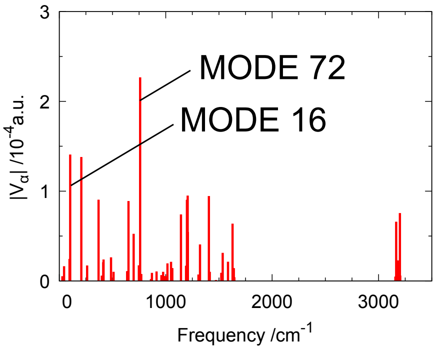

(b) **(c)**

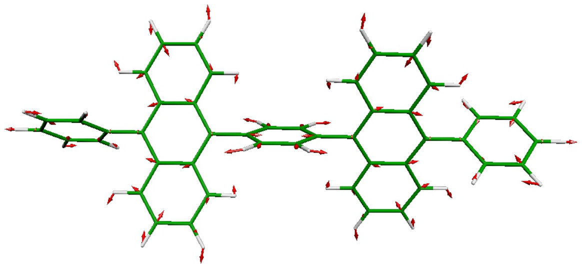

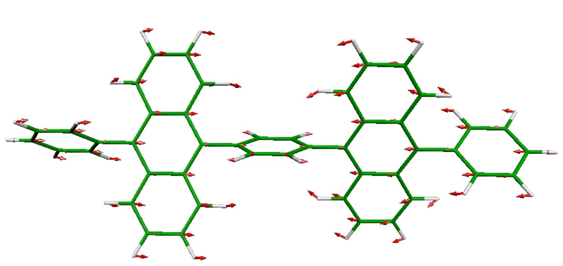

Figure S8: (a) Off-diagonal VCCs between S_2 and S_0 at the S_2 optimized structure, and strong coupling modes: (b) mode 72 and (c) mode 16.

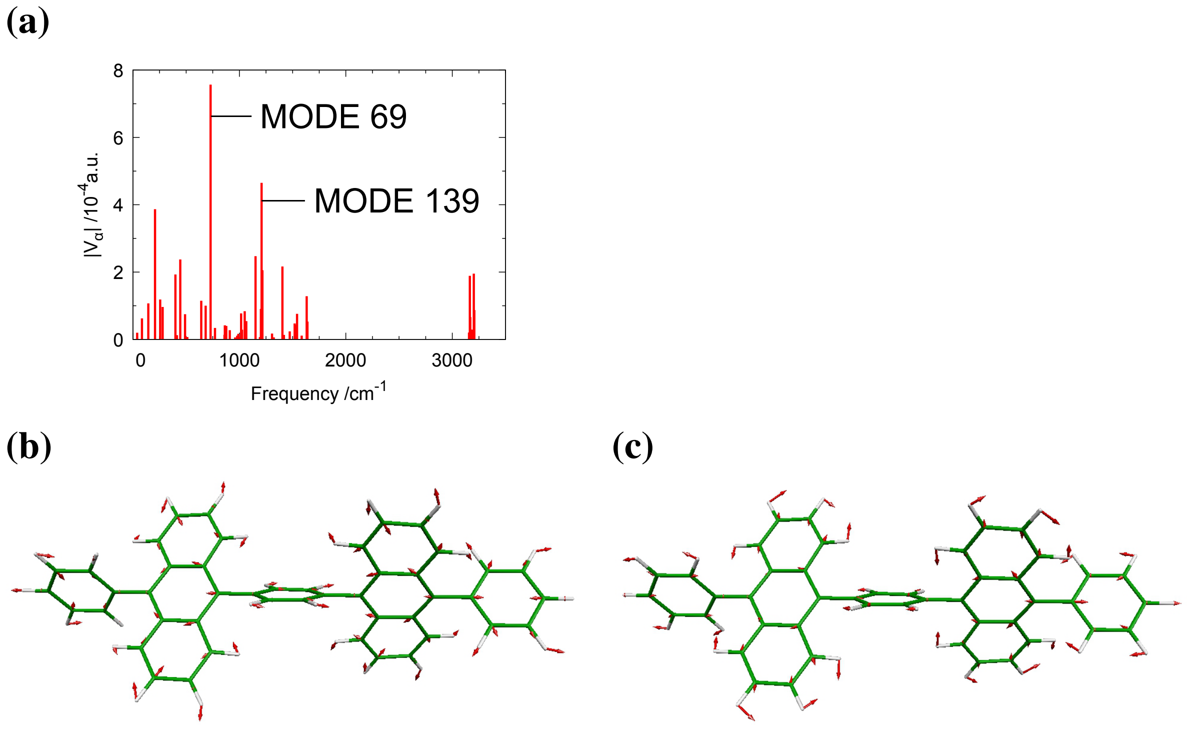

Figure S9: (a) Off-diagonal VCCs between S_1 and S_0 at the S_1 optimized structure, and strong coupling modes: (b) mode 69 and (c) mode 139.

S9 Vibronic Coupling Density Analyses

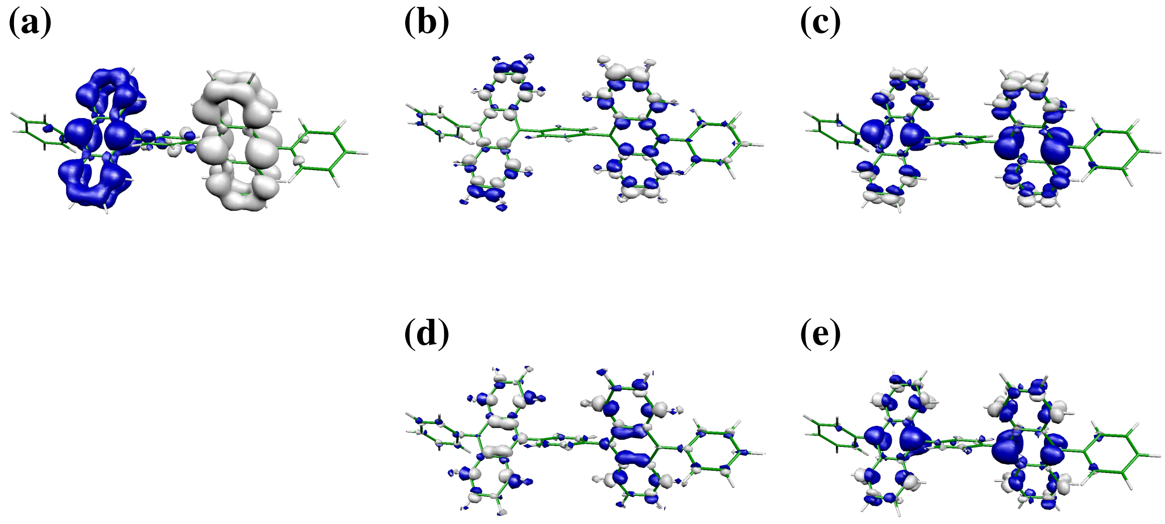

Figure S10: Off-diagonal VCD analyses between T_3 and T_4 at the T_3 optimized structure: (a) overlap density ρ_{34}, (b) potential derivative v_{33}, (c) VCD $\eta_{34,33}$, (d) potential derivative v_{64}, and (e) VCD $\eta_{34,64}$. The isosurface values for ρ, v, and η are 1.0×10^{-3}, 5.0×10^{-3} and 5.0×10^{-6} a.u., respectively.

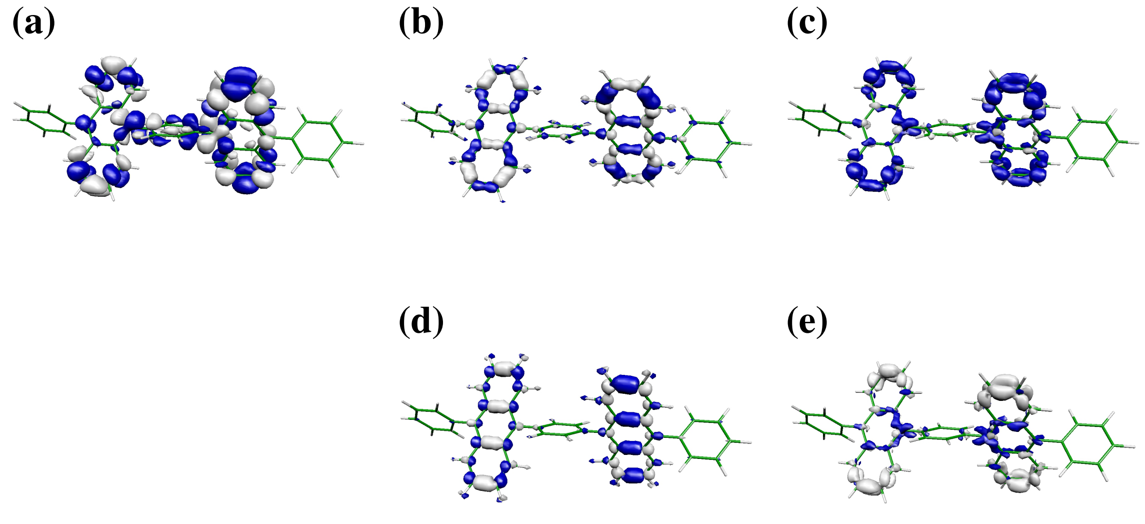

Figure S11: Off-diagonal VCD analyses between T_4 and T_2 at the T_4 optimized structure: (a) overlap density ρ_{42}, (b) potential derivative v_{163}, (c) VCD $\eta_{42,163}$, (d) potential derivative v_{183}, and (e) VCD $\eta_{42,183}$. The isosurface values for ρ, v, and η are 1.0×10^{-4}, 5.0×10^{-3} and 5.0×10^{-7} a.u., respectively.

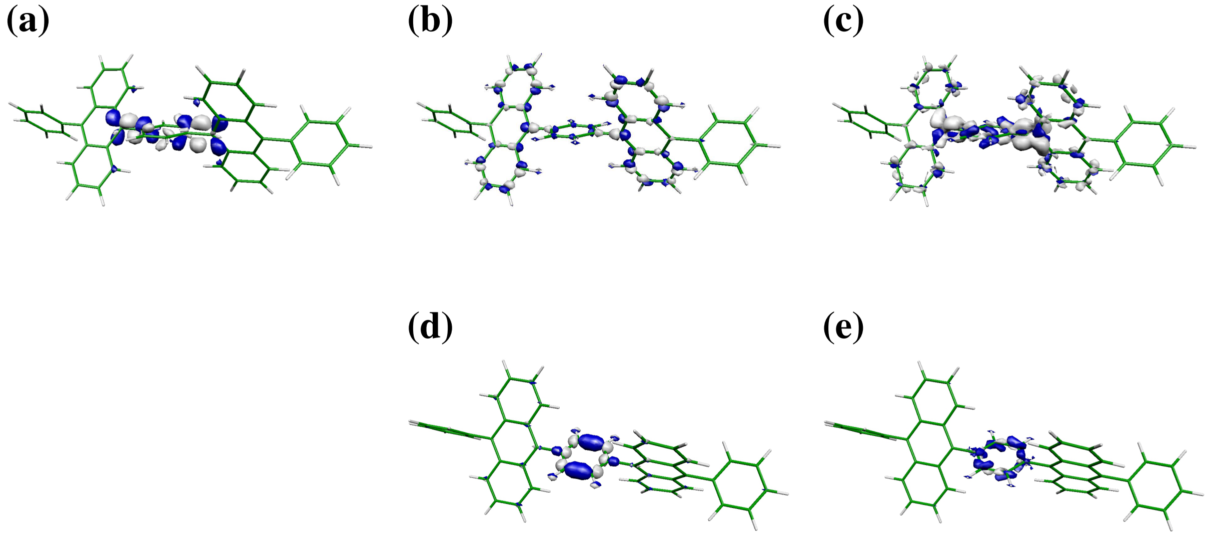

Figure S12: Off-diagonal VCD analyses between T_4 and T_1 at the T_4 optimized structure: (a) overlap density ρ_{41}, (b) potential derivative v_{164}, (c) VCD $\eta_{41,164}$, (d) potential derivative v_{192}, and (e) VCD $\eta_{41,192}$. The isosurface values for ρ, v, $\eta_{41,164}$, and $\eta_{41,192}$ are 5.0×10^{-4}, 1.0×10^{-2}, 1.0×10^{-6}, and 5.0×10^{-6} a.u., respectively.

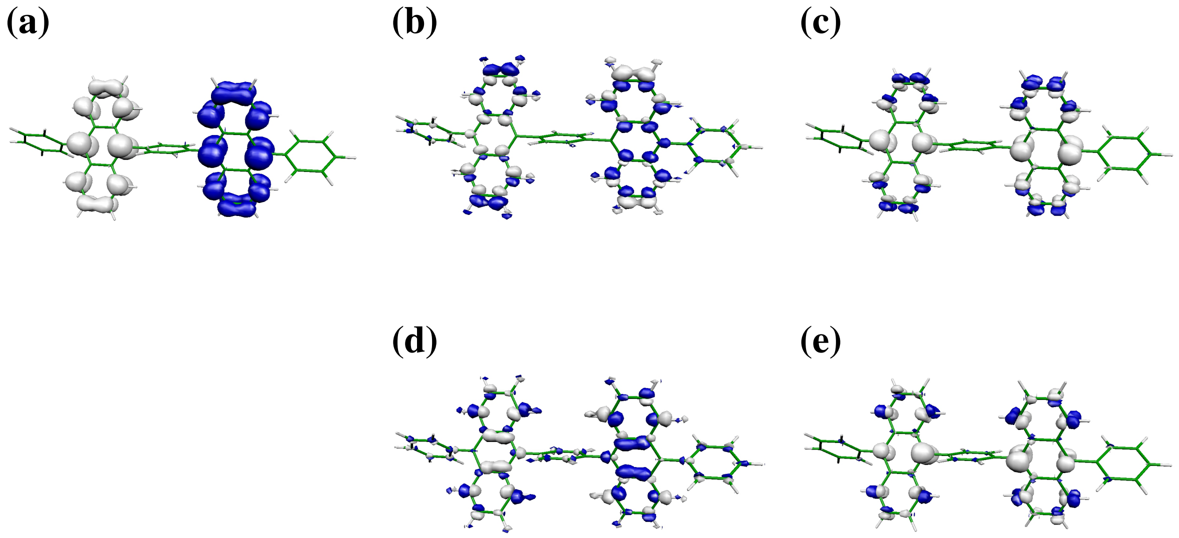

Figure S13: Off-diagonal VCD analyses between S_2 and S_1 at the S_2 optimized structure: (a) overlap density ρ_{21}, (b) potential derivative v_{33}, (c) VCD $\eta_{21,33}$, (d) potential derivative v_{64}, and (e) VCD $\eta_{21,64}$. The isosurface values for ρ, v, and η are 1.0×10^{-3}, 5.0×10^{-3} and 5.0×10^{-6} a.u., respectively.

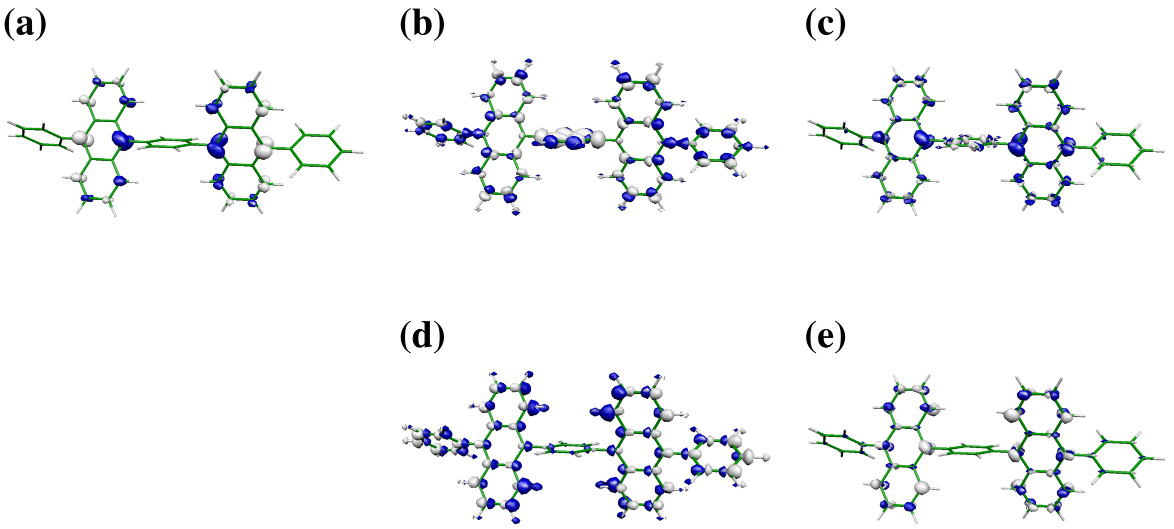

Figure S14: Off-diagonal VCD analyses between S_2 and S_0 at the S_2 optimized structure: (a) overlap density ρ_{20}, (b) potential derivative v_{72}, (c) VCD $\eta_{20,72}$, (d) potential derivative v_{16}, and (e) VCD $\eta_{20,16}$. The isosurface values for ρ, v, and η are 1.0×10^{-3}, 5.0×10^{-3} and 3.0×10^{-6} a.u., respectively.

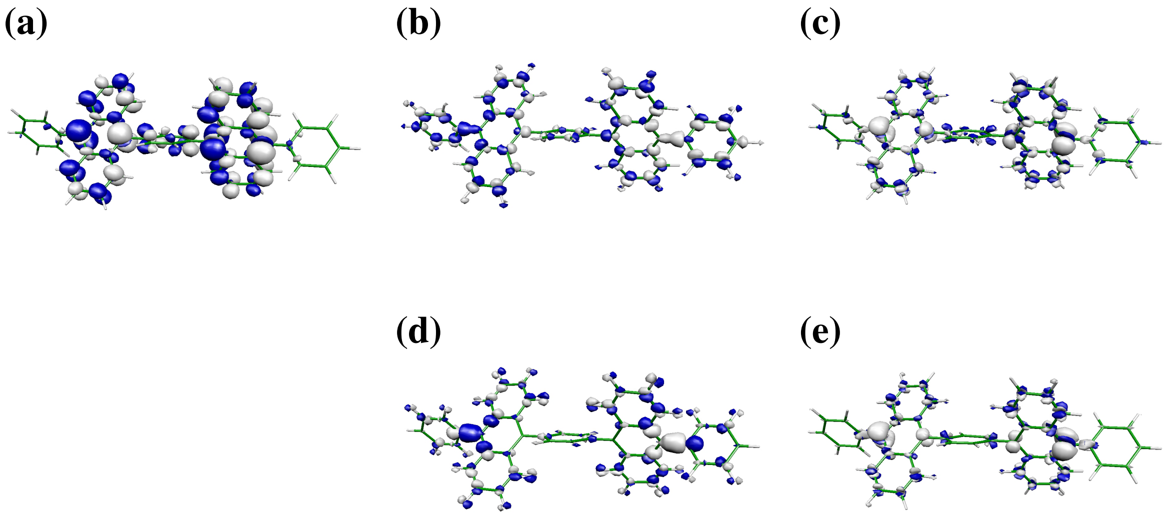

Figure S15: Off-diagonal VCD analyses between S_1 and S_0 at the S_1 optimized structure: (a) overlap density ρ_{10}, (b) potential derivative v_{69}, (c) VCD $\eta_{10,69}$, (d) potential derivative v_{139}, and (e) VCD $\eta_{10,139}$. The isosurface values for ρ, v, and η are 1.0×10^{-3} 5.0×10^{-3}, and 5.0×10^{-6} a.u., respectively.

S10 Orbital Overlap Densities

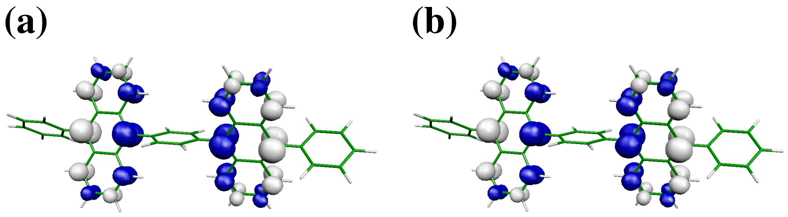

Figure S16: Orbital overlap densities, (a) $\psi_{\mathrm{HO}}\psi_{\mathrm{NLU}}$ and (b) $\psi_{\mathrm{NHO}}\psi_{\mathrm{LU}}$, at the optimized structure for S_2. The isosurface value is 1.0×10^{-3} a.u.

S11 General Conditions for Disappearances of Overlap Densities in Pseudo-Degenerate Systems

In this section, we discuss the overlap densities in a pseudo-degenerate system, (moiety L)-(linker)-(moiety R) system, in general, where moiety L and moiety R are the same fragments. As the pseudo-degenerate frontier orbitals, we consider the following forms:

$$\psi_{\mathrm{LU}} = \frac{1}{\sqrt{2}}(\phi_{\mathrm{LU}}(L) - \phi_{\mathrm{LU}}(R)), \qquad \psi_{\mathrm{NLU}} = \frac{1}{\sqrt{2}}(\phi_{\mathrm{LU}}(L) + \phi_{\mathrm{LU}}(R)), \tag{S17}$$

$$\psi_{\mathrm{HO}} = \frac{1}{\sqrt{2}}(\phi_{\mathrm{HO}}(L) + \phi_{\mathrm{HO}}(R)), \qquad \psi_{\mathrm{NHO}} = \frac{1}{\sqrt{2}}(\phi_{\mathrm{HO}}(L) - \phi_{\mathrm{HO}}(R)), \tag{S18}$$

where $\phi_{\mathrm{HO/LU}}(L/R)$ denotes a fragment HOMO/LUMO on moiety Left(L)/Right(R).

Since these frontier orbitals are localized on moieties L and R, the overlaps between the different moieties can be neglected: $\phi_i(L)\phi_j(R) = 0$ ($i, j =$HO, NHO, LU, and NLU). Therefore, we can obtain the following relations of orbital overlap densities:

$$|\psi_{\mathrm{LU}}|^2 = \frac{1}{2}(|\phi_{\mathrm{LU}}(L)|^2 + |\phi_{\mathrm{LU}}(R)|^2) = |\psi_{\mathrm{NLU}}|^2 =: r, \tag{S19}$$

$$\psi_{\mathrm{HO}}\psi_{\mathrm{NLU}} = \frac{1}{2}(\phi_{\mathrm{LU}}(L)\phi_{\mathrm{HO}}(L) + \phi_{\mathrm{LU}}(R)\phi_{\mathrm{HO}}(R)) = \psi_{\mathrm{NHO}}\psi_{\mathrm{LU}} =: s, \tag{S20}$$

$$|\psi_{\mathrm{HO}}|^2 = \frac{1}{2}(|\phi_{\mathrm{HO}}(L)|^2 + |\phi_{\mathrm{HO}}(R)|^2) = |\psi_{\mathrm{NHO}}|^2 =: t, \tag{S21}$$

$$\psi_{\mathrm{HO}}\psi_{\mathrm{LU}} = \frac{1}{2}(\phi_{\mathrm{LU}}(L)\phi_{\mathrm{HO}}(L) - \phi_{\mathrm{LU}}(R)\phi_{\mathrm{HO}}(R)) = \psi_{\mathrm{NHO}}\psi_{\mathrm{NLU}} =: u. \tag{S22}$$

In addition, we define the orbital overlap densities

$$q := \psi_{\mathrm{HO}}\psi_{\mathrm{NHO}} = \frac{1}{2}(|\phi_{\mathrm{HO}}(L)|^2 - |\phi_{\mathrm{HO}}(R)|^2), \tag{S23}$$

$$p := \psi_{\mathrm{LU}}\psi_{\mathrm{NLU}} = \frac{1}{2}(|\phi_{\mathrm{LU}}(L)|^2 - |\phi_{\mathrm{LU}}(R)|^2). \tag{S24}$$

We consider wave functions of the pseudo-degenerate system shown in TABLE S10. The overlap densities of the pseudo-degenerate system are summarized in TABLE S11. The pairs denoted by 0 in TABLE S11 indicate reduced overlap density, i.e. suppressed radiative/non-radiative transition. This approach can be extended for degenerate systems and pseudo-degenerate systems with more than three-fold degeneracy.

Table S10: Approximate wave functions of a pseudo-degenerate system, $c = 1/\sqrt{2}$.

Approximate form of wave function	@T_4/@T_3	@S_2/@S_1
$\Psi_a = c\Phi_{HO}^{LU} - c\Phi_{NHO}^{NLU}$	T_4	
$\Psi_b = c\Phi_{HO}^{NLU} - c\Phi_{NHO}^{LU}$	T_3	S_2
$\Psi_c = c\Phi_{HO}^{LU} + c\Phi_{NHO}^{NLU}$	T_1	
$\Psi_d = \Phi_{HO}^{LU}$		S_1
$\Psi_e = c\Phi_{HO}^{NLU} + c\Phi_{NHO}^{LU}$	T_2	S_3
$\Psi_f = \Phi_{HO}^{NLU}$		
$\Psi_g = \Phi_{NHO}^{NLU}$		S_4
$\Psi_h = \Phi_{NHO}^{LU}$		
$\Psi_0 = \Phi_0$		S_0

Table S11: Overlap densities in a pseudo-degenerate system. $\alpha = p - q$, $\beta = \rho_0 - t + r$, and $\gamma = p + q$.

	Ψ_a	Ψ_b	Ψ_c	Ψ_d	Ψ_e	Ψ_f	Ψ_g	Ψ_h	Ψ_0
Ψ_a		α	0	$c\beta$	0	$c\alpha$	$-c\beta$	$-c\alpha$	0
Ψ_b			0	$c\alpha$	0	$c\beta$	$-c\alpha$	$-c\beta$	0
Ψ_c				$c\beta$	γ	$c\gamma$	$c\beta$	$c\gamma$	$2cu$
Ψ_d					$c\gamma$	p	0	q	u
Ψ_e						$c\beta$	$c\gamma$	$c\beta$	$2cs$
Ψ_f							q	0	s
Ψ_g								p	u
Ψ_h									s
Ψ_0									

References

1 Sato, T. *et al.* Vibronic coupling density and related concepts. *J. Phys.: Conf. Ser.* **428**, 012010 1–19 (2013).

2 Uejima, M., Sato, T., Yokoyama, D., Tanaka, K. & Park, J.-W. Quantum yield in blue-emitting anthracene derivatives: vibronic coupling density and transition dipole moment density. *Phys. Chem. Chem. Phys.* **16**, 14244–14256 (2014).
